# Dermatoscopy and Optical Coherence Tomography in Vulvar High-Grade Squamous Intraepithelial Lesions and Lichen Sclerosus: A Prospective Observational Trial

**DOI:** 10.1097/LGT.0000000000000731

**Published:** 2023-03-16

**Authors:** Bertine W. Huisman, Lisa Pagan, Rosanne G.C. Naafs, Wouter ten Voorde, Robert Rissmann, Jurgen M.J. Piek, Jeffrey Damman, Maria J. Juachon, Michelle Osse, Tessa Niemeyer-van der Kolk, Colette L.M. van Hees, Mariette I.E. van Poelgeest

**Affiliations:** 1 Centre for Human Drug Research, Leiden, The Netherlands; 2 Department of Obstetrics and Gynaecology, Leiden University Medical Center, Leiden, The Netherlands; 3 Leiden Academic Centre for Drug Research, Leiden University, Leiden, The Netherlands; 4 Department of Obstetrics and Gynaecology and Catharina Cancer Institute, Catharina Ziekenhuis, Eindhoven, The Netherlands; 5 Department of Pathology, Erasmus MC, University Medical Center, Rotterdam, The Netherlands; 6 Department of Dermatology, Erasmus Medical Centre, Rotterdam, The Netherlands

**Keywords:** dermatoscopy, D-OCT, lichen sclerosus, vulvar HSIL

## Abstract

This study aimed to examine potential discriminatory characteristics of dermatoscopy and dynamic optical coherence tomography (D-OCT) on vulvar high-grade squamous intraepithelial lesions (vHSIL) and lichen sclerosus (LS) compared with healthy vulvar skin.

**Methods:**

A prospective observational clinical trial was performed in 10 healthy volunteers, 5 vHSIL and 10 LS patients. Noninvasive imaging measurements using dermatoscopy and D-OCT were obtained at several time points, including lesional and nonlesional vulvar skin. Morphologic features of vHSIL and LS were compared with healthy controls. Epidermal thickness and blood flow were determined using D-OCT. Patients reported tolerability of each study procedure, including reference vulvar biopsies. The main outcome measures were feasibility and tolerability of imaging modalities, dermatoscopy and OCT characteristics, OCT epidermal thickness and D-OCT dermal blood flow.

**Results:**

The application of dermatoscopy and D-OCT is feasible and tolerable. In vHSIL, dermatoscopic warty structures were present. In LS, sclerotic areas and arborizing vessels were observed. Structural OCT in the vulvar area aligned with histology for hyperkeratosis and dermal-epidermal junction visualization. Currently, the OCT algorithm is unable to calculate the epidermal thickness of the uneven vulvar area. Dynamic optical coherence tomography showed statistically significant increased blood flow in LS patients (mean ± SD, 0.053 ± 0.029) to healthy controls (0.040 ± 0.012; *p* = .0024).

**Conclusions:**

The application of dermatoscopy and D-OCT is feasible and tolerable in vHSIL and LS patients. Using dermatoscopy and D-OCT, the authors describe potential characteristics to aid differentiation of diseased from healthy vulvar skin, which could complement clinical assessments.

## Abstract 2

This feasibility study presents dermatoscopy and dynamic optical coherence tomography as easy-to-use, well-tolerated and noninvasive imaging tools aiding recognition of vulvar high-grade intraepithelial lesions and lichen sclerosus.

Inadequate clinical recognition and delayed or inadequate treatment of vulvar high-grade intraepithelial lesions (vHSIL) and lichen sclerosus (LS) can have considerable physical, sexual, and psychological impact.^1^ The diagnostic process and disease follow-up of these vulvar diseases should be improved by objective and noninvasive, disease-specific biomarkers.^2^ A prerequisite in biomarker validation is its ability to discriminate healthy from diseased tissue.^3^

Examples of potential novel techniques are dermatoscopy and dynamic optical coherence tomography (D-OCT). Dermatoscopy is routinely applied by dermatologists as an adjunctive tool to ameliorate subsurface structure visualization and pattern identification to aid diagnosis of melanoma, basal cell carcinoma, and other cutaneous disorders.^4^ Its application on the vulvar area is currently limited to research purposes of vulvar pigmented lesions.^5–7^ Dynamic optical coherence tomography is a noninvasive imaging technique that provides real-time cross-sectional images of biological structures. It has been incorporated in the daily ophthalmology practice for diagnosis of retinal diseases.^8^ Dynamic optical coherence tomography has been applied as a research tool in dermatology for characterization of nonmelanoma skin cancer.^9,10^ In gynecology, a few studies in cervical, vulvar, and ovarian tissue suggest potential for D-OCT to differentiate between healthy and premalignant tissue of epithelial origin.^11–14^

The study objective was to examine and describe potential discriminatory characteristics of dermatoscopy and D-OCT on premalignant vulvar skin compared with healthy vulvar skin. Therefore, we examined dermatoscopy and D-OCT on vHSIL and LS patients and healthy controls.

## METHODS

A prospective, healthy volunteer-matched, single-center trial conducted at the Centre for Human Drug Research, Leiden, The Netherlands, was performed from February to October 2021. The Declaration of Helsinki was the guiding principle for trial execution. The study was approved by an independent medical ethics committee “Medisch-Ethische Toetsingscommissie Leiden Den Haag Delft” and registered in “Nederlands Trial Register” (NL73964.058.20) and “EudraCT” (2020–002201-2). All subjects provided written informed consent before participation. These imaging results are part of a multimodal pilot study investigating research methods to identify biomarkers that could improve vulvar disease identification and therapeutic response monitoring (Figure S1, http://links.lww.com/LGT/A297).^15^

### Study Design and Subjects

In total, 25 women, aged 25 to 95 years, with a body mass index (BMI) less than 30 kg/m^2^ were included: 5 vHSIL patients (≥1 sharply margined histologically confirmed vHSIL lesion ≥15 mm), 10 LS patients (clinical and/or histological diagnosis confirmation), and 10 healthy controls (confirmed absence of vulvar disease). Main exclusion criteria were significant other diseases, pregnancy, other vulvar conditions, immunocompromised state, sexually transmitted disease, AIDS, or hepatitis. For standardization, washout for topically applied products on the vulvar area was 14 days or more.^16^

All subjects visited the clinical research department on day 0 (0 h = baseline, 3 h, and 6 h), day 1, and day 7 (Figure S2, http://links.lww.com/LGT/A298). The LS patients also visited the clinic on days 21 and 35 as follow-up for a 4-week standard-of-care treatment with corticosteroid ointment clobetasol 0.05% (Dermovate, GlaxoSmithKline, Brentford, United Kingdom) starting at day 8. At each visit, clinical assessments and noninvasive imaging measurements were performed. Biopsies were obtained at day 0 for all subjects and on day 35 for LS patients.

### Anogenital Examination

Examination of the anogenital region and study procedures were performed in a gynecological chair. All patients were assessed by trained physicians (B.H. and L.P.) and discussed with an oncological gynecologist specialized in vulvar disease (M.v.P.) at baseline. Although LS has a heterogeneous clinical presentation, we opted out of further stratification due to low patient numbers and the exploratory scope of our study. All procedures were performed on selected target areas, including a lesional and nonlesional site for all patients.

### Imaging

#### Dermatoscopy on the Vulvar Area

Macroscopic dermatoscopic images of the vulvar surface were obtained using a FotoFinder Medicam 1000 with the Bodystudio ATBM (FotoFinder Systems GmbH, Bad Birnbach, Germany) for photo analysis and documentation (Figure S3, http://links.lww.com/LGT/A299). Microscopic images were obtained with a D-Scope IV (FotoFinder systems, GmbH, Bad Birnbach, Germany) dermatoscopy lens with polarized light and analyzed using FotoFinder universe. Dermatoscopy includes a follow-up photo documentation function.

##### Microscopic characteristics

Individual dermatoscopic characteristic or a set of characteristics were exploratorily assessed for discriminatory potential for vulvar diseases. An expert dermatologist fully blinded for patient type (C.H.) scored characteristics in decoded microscopic images. The characteristics included: color of the skin (red, pink, yellow, gray, brown, white, or other), vessel density or concentration (increased, normal, decreased, or invisible), and vessel concentration (dotted, hairpin, linear, linear serpentine, thin/thick arborizing, thick root-like, other, or not visible). The presence or absence of scales, ecchymoses, purpura, yellow-white structureless areas, white circles, peppering, comedo-like openings, ulceration, and warty structures were reported, as previously described in the literature of dermatoscopy characteristics of genital lesions.^17,18^ In total, 85 photos were scored (15 vHSIL, 40 LS, and 30 healthy volunteers) obtained at days 0, 7, and 35.

#### D-Oct

Skin morphology analysis up to a depth of 1 mm was performed by D-OCT using the Vivosight Dx (Michelson Diagnostics Ltd, United Kingdom) (Figure S4, http://links.lww.com/LGT/A300). Scans with artifacts due to movement were excluded from analysis and directly retaken. Data were stored and analyzed using VivoSight and VivoTools version 4.15. Qualitative assessments were performed by 3 trained OCT operators (B.H., L.P., and W.V.).

##### Epidermal thickness

Epidermal thickness was determined using algorithms incorporated in the software. Manual epidermal thickness analyses were performed with ImageJ (version Java 1.8.0_172, Bethesda, MD). Using a self-generated macro, 3 consecutive vertical lines were drawn for the epidermal layer per scan. The mean, SD, and minimum and maximum epidermal thickness were determined per set of 120 consecutive scans per patient. Baseline and posttreatment (LS) scans were analyzed manually for epidermal thickness due to the exploratory and time-consuming nature of manual calculations.

##### Blood flow

The D-OCT blood flow was determined using the algorithms incorporated in the software. The quantification of the blood flow was based on the average speckle signal returning at the detector at dermal depth from 0.10 to 0.35 mm to reduce contortions from artifacts.^19^

### Histological Analysis

Vulvar tissue samples were obtained using a 4-mm punch biopsy acquired by trained physicians (B.H., L.P., and M.v.P.) at the end of day 0. The skin was anesthetized using subcutaneous lidocaine before the procedure. The obtained biopsies were stained for hematoxylin and eosin by the Erasmus Medical Centre (EMC, Rotterdam, The Netherlands) following clinical protocols. Slides were scored by a dermatopathologist (J.D.). Dysplasia was assessed by the epidermal levels of atypia and scored as warty and/or basaloid types. Lichen sclerosus was diagnosed by histological characteristics.^20,21^ Inno-LiPa HPV Genotyping Extra (Eurofins NMDL-LCPL, Rijswijk, The Netherlands) was used for human papillomavirus (HPV) typing.^22^

### Patient Reported Outcomes

The “burdensome questionnaire” comprised 100-mm lines that the patient completed for each study procedure ranging from 0 mm, “no burden at all”, to 100 mm, “the most burdensome procedure possible”. The e-diary (Promasys ePRO platform) with a reminder and photograph function (with corresponding timestamps) monitored at-home drug compliance.

### Statistical Analysis

Dermatoscopic observations were summarized and shown descriptively. Differences of D-OCT between patient groups were tested using Mann-Whitney *U* test on baseline averages of 2 groups (vHSIL, LS, or healthy). A paired, 2-tailed *t* test was performed comparing mean D-OCT values pretreatment to posttreatment. Differences for the burdensome questionnaire were analyzed using a paired Student *t* test comparing dermatoscopy and D-OCT to the biopsy procedure. The analyses were computed in SAS 9.4 (SAS Institute, Cary, NC) and GraphPad version 9.3.1 (GraphPad Software, Inc, San Diego, CA).

## RESULTS

In total, 25 women (5 patients with vHSIL, 10 patients with LS, and 10 healthy controls) were enrolled and finished the study (Table S1, http://links.lww.com/LGT/A304). Fitzpatrick skin type ranged from I to III. Menopausal status was equally distributed among groups.

### Dermatoscopy

Vulvar skin of a representative subject of each cohort (vHSIL, LS, and healthy controls) captured by dermatoscopy is presented in a macroscopic overview and a microscopic image (Figure S5, http://links.lww.com/LGT/A301).

#### Microscopic Characteristics

**FIGURE 1:**
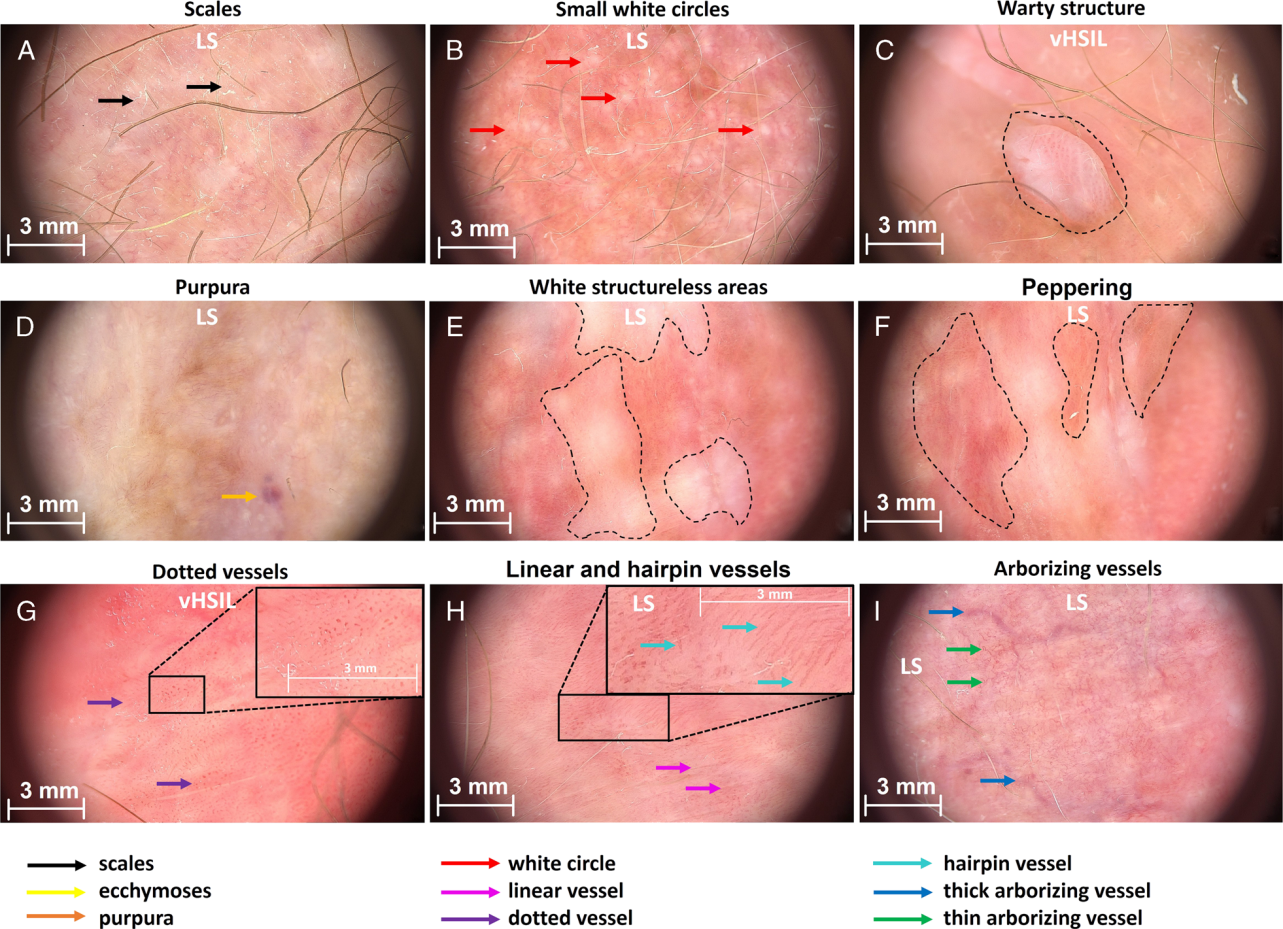
Representative images showing examples of scored characteristics. A, Scales (LS). B, Small white circles (LS). C, Warty structure (vHSIL). D, Purpura (LS). E, White structureless areas (LS). F, Peppering (healthy vulva). G, Dotted vessels (vHSIL). H, Linear and hairpin vessels (LS). I, Thick and thin arborizing vessels (LS).

**FIGURE 2:**
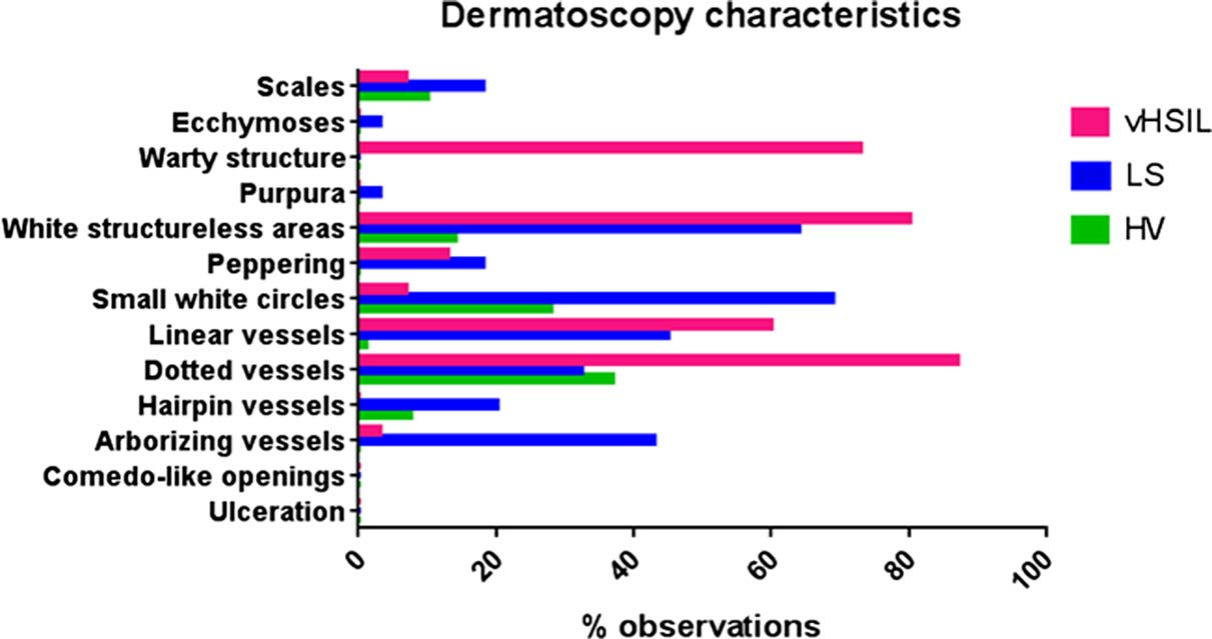
Percentage of observed characteristics per patient group (85 photos were scored: 15 vHSIL, 40 LS, and 30 healthy volunteers) from the scoring of microscopic dermatoscopy images by a blinded dermatologist (C.H.). HV, healthy vulva.

Examples of dermatoscopic characteristics are shown in Figure 1 and the frequency of observations per group are summarized in Figure 2. The most prominent characteristic of vHSIL were warty structures (4/5), which could be accompanied by some scales and peppering. The color of vHSIL skin was highly variable. Women with Fitzpatrick skin type higher than III could not be recruited, so color findings may vary based on the analyzed population. Vessels were present with dotted or linear vessel. Lichen sclerosus typically showed white structureless areas (8/10) and/or increased vessel concentration (8/10), with arborizing and/or thick root-like vessel morphology. The vulvar skin of healthy controls was mostly yellow (8/10), with normal vessel pattern of dotted or linear vessels sometimes accompanied by white circles (4/10). Occasionally, white structureless areas or peppering was observed (3/10). No changes were observed in LS skin after a 4-week clobetasol treatment (data not shown).

### D-Oct

#### Morphological Characteristics

**FIGURE 3:**
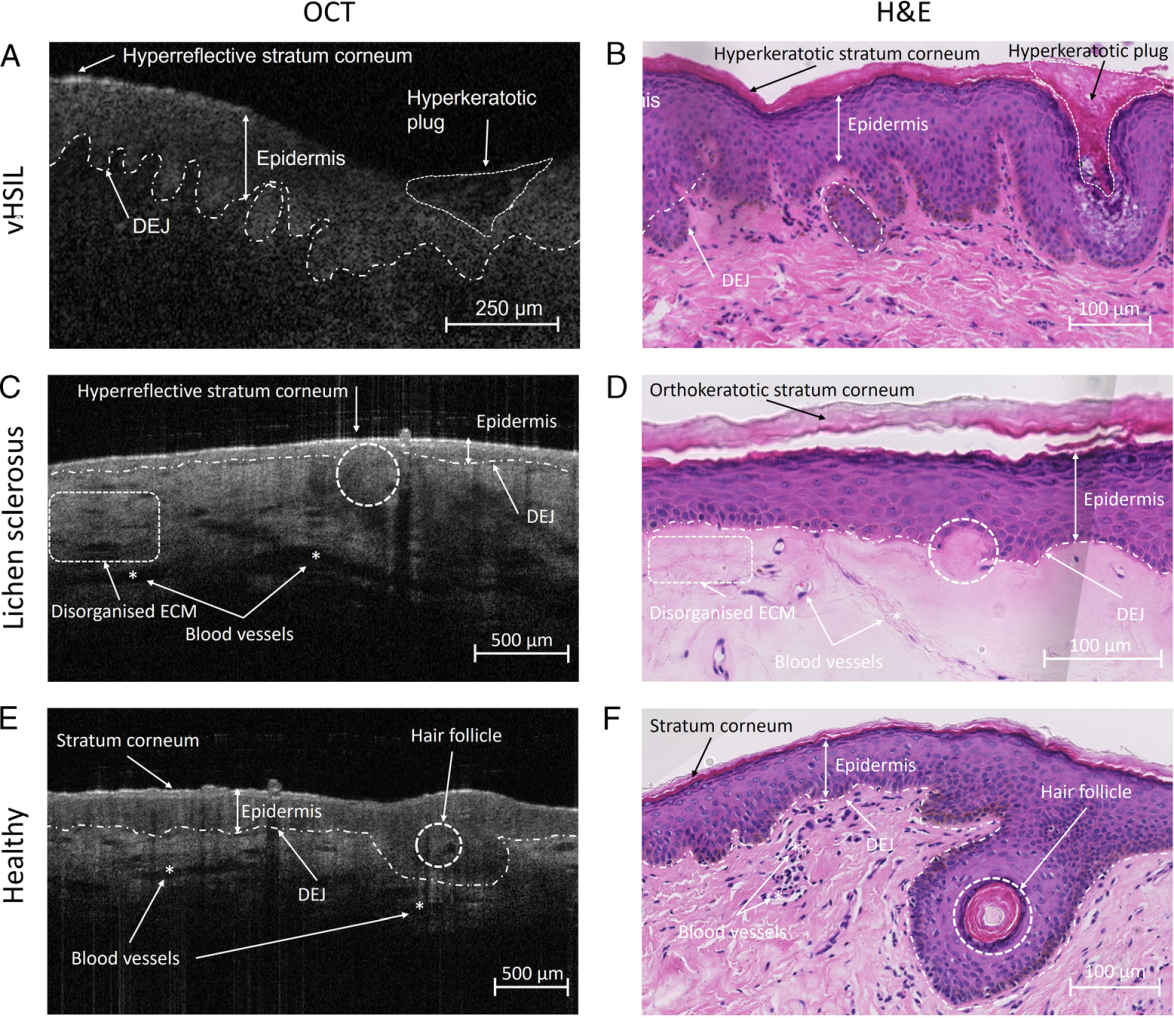
Structural OCT recordings compared with aligned histological assessments of A + B, vHSIL, (C + D) lichen sclerosus, and (E + F) healthy volunteers. Asterisks (*) indicate blood vessels. DEJ, dermal-epidermal junction; ECM, extracellular matrix; H&E, hematoxylin and eosin.

Vulvar high-grade squamous intraepithelial lesions is histologically characterized by hyperkeratosis and parakeratosis, acanthosis with club-shaped rete ridges, cytonuclear atypia, disorientation of individual epithelial cells, and an intact basement membrane.^23^ Hyperkeratosis could be identified in the structural OCT image of a vHSIL lesion as hyperreflective stratum corneum. The OCT shows the broadening of the epidermis in the club-shaped pattern associated with acanthotic vHSIL with an intact dermal-epidermal junction, as observed in histology (Figure 3A). Lichen sclerosus is histologically characterized by hyperkeratosis, epidermal thinning with loss of the rete ridge pattern, and dermal changes, including sclerosis.^20,21^ These changes can also be identified with OCT, especially the disorganized extracellular matrix reflecting dermal changes (Figure 3B). Nuclear and cellular changes cannot be visualized using OCT. We observed that resolution was occasionally lost under a hyperkeratotic and hyperreflective stratum corneum or sclerotic area. These OCT findings are pronounced in vHSIL and LS compared with healthy vulvar skin. Histologically, healthy vulvar skin has a normal epidermal thickness in the absence of characteristics observed in diseased vulvar skin. These features could also be visualized in OCT recordings (Figure 3C).

#### Epidermal Thickness

**FIGURE 4:**
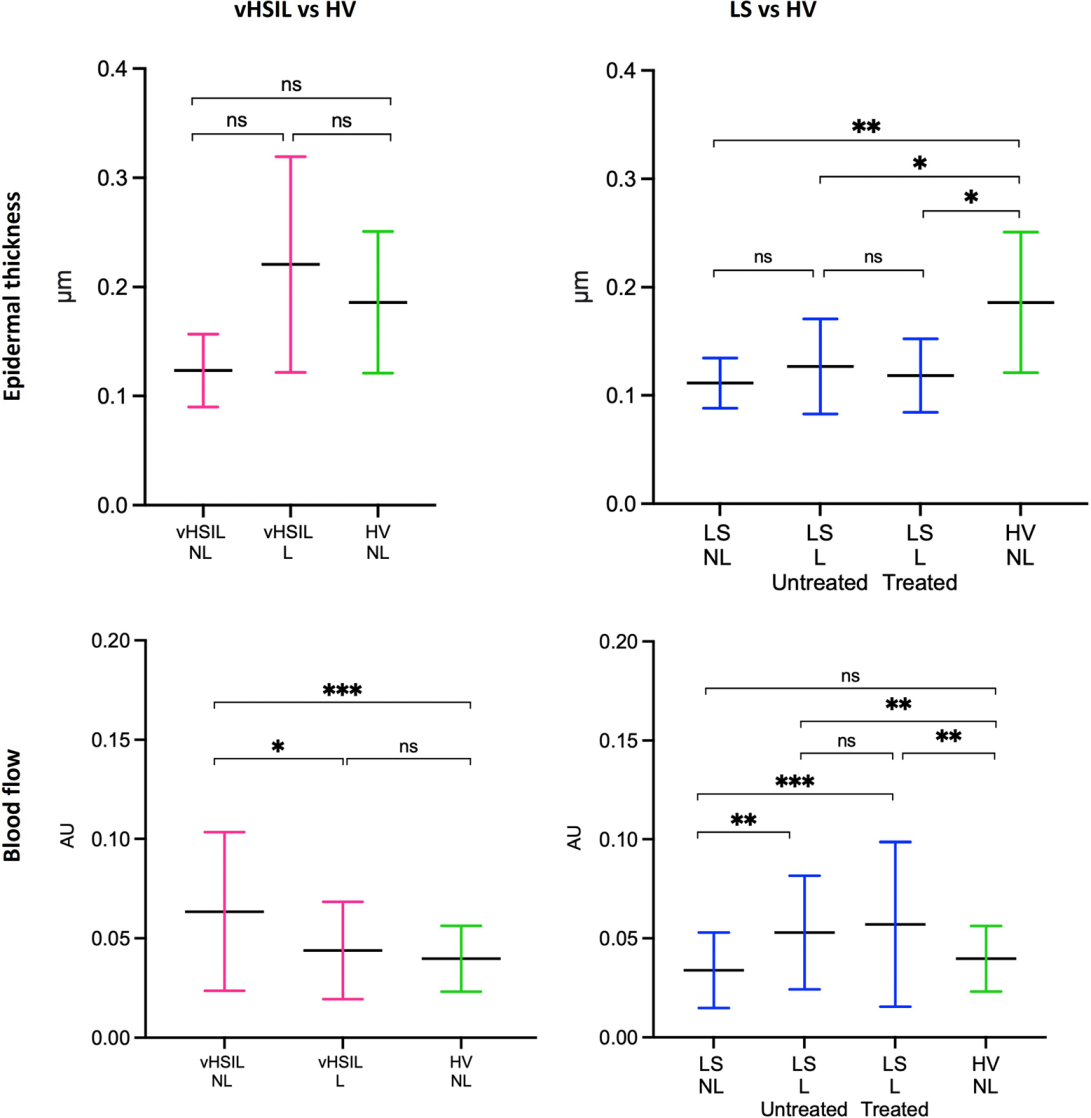
The D-OCT epidermal thickness measurements (manually determined using ImageJ) and blood flow measurements (determined by incorporated algorithm). Measurements were assessed at baseline (D1H0) and, for LS only, posttreatment (D35). The mean and SDs are displayed for each group. A, Epidermal thickness in μm (*y* axis) is plotted against measurements clustered per patient group (*x* axis). B, The average blood flow measured between a skin depth of 0.10–0.35 μm at nonbiopsy sites of vHSIL and LS subjects compared with healthy controls. The mean and SDs are displayed for each group as well as individual data points. Blood flow in AU (*y* axis) plotted against measurements clustered per patient group (*x* axis). ns = *p* > .05, * = *p* ≤ .05, ** = *p* ≤ .01, *** = *p* ≤ .001, **** = *p* ≤ .0001. L, lesional skin; NL, nonlesional skin.

In total, 77.5% (vHSIL), 56.9% (LS), and 91.2% (healthy control) of the measurements using the incorporated algorithm failed because an impossible epidermal thickness of 0 μm was reported. Therefore, manual epidermal thickness measurements were performed (Figure 4A+B). No significant differences in epidermal thickness were identified comparing lesional or nonlesional vHSIL to healthy controls. The epidermis (mean ± SD) of preclobetasol lesional LS (0.13 ± 0.10 μm) was significantly thinner compared with healthy controls (0.19 ± 0.06 μm), *p* = .0312. No differences were observed between preclobetasol and postclobetasol-treated LS (0.127 ± 0.10 μm vs 0.118 ± 0.034; *p* = .643).

#### Blood Flow

At baseline, higher blood flow (mean ± SD) was observed in nonlesional vHSIL (0.063 ± 0.040) compared with lesional vHSIL (0.044 ± 0.025), *p* = .0255 (Figure 4C). No differences were detected between lesional vHSIL and healthy controls (0.040 ± 0.017; *p* = .347). Blood flow in nonlesional vHSIL skin differed significantly from healthy controls (0.063 ± 0.040; *p* = .0001). Blood flow was significantly higher in pretreatment lesional LS (0.053 ± 0.029) compared with nonlesional LS (0.034 ± 0.019; *p* < .0001) and healthy controls (0.040 ± 0.012; *p* = .0024) (Figure 4D). Nonlesional LS did not differ significantly compared with healthy controls (*p* = .077). No differences were observed between preclobetasol and postclobetasol-treated LS (0.057 ± 0.042) (*p* = .532). Blood flow measurements fluctuated over time (days 0, 2, 8, 22, and 36), depending on sample location (lesional vs nonlesional) (Figure S6, http://links.lww.com/LGT/A302).

### Histological Analysis

All clinical diagnoses of nonlesional and lesional skin of vHSIL, nonlesional LS, and healthy controls were confirmed in biopsy (Table S2, http://links.lww.com/LGT/A305). The biopsies of lesional LS were classified as LS in 3/10 cases. The remaining were classified as normal vulvar skin with inflammatory reactive changes, although clinical LS diagnosis had been confirmed by a specialized gynecologist (M.v.P.) before enrolment. Positivity for HPV16 was identified in 4/5 lesional vHSIL biopsies. One lesional LS biopsy tested positive for HPV type 53, whereas no HPV was found in nonlesional or healthy control biopsies.

### Patient-Friendliness and Treatment Compliance

All imaging methods applied in this study were considered mildly burdensome and therefore patient friendly. Vulvar biopsy was considered substantially more burdensome than all noninvasive imaging procedures, with mean scores greater than 20 mm (Figure S7, http://links.lww.com/LGT/A303). Treatment compliance was 99%.

## DISCUSSION

This exploratory study shows that application of dermatoscopy and D-OCT is feasible and tolerable in vHSIL and LS patients. The most prominent finding with dermatoscopy was the presence of sclerotic areas and arborizing vessels in LS and warty structures for vHSIL. Structural OCT images could be aligned for both diseases with histology. A novel finding in this study was the increased blood flow measured by the algorithm of the D-OCT in vulvar LS compared with healthy tissue. Epidermal thickness determination by OCT should be considered for research purposes only at this stage.

The main strength is the prospective trial design in vulvar patients and healthy controls including within-subject lesional and nonlesional control. Including only within-patient “healthy” control sites can be invalid because the vulvar tissue may be affected by scarring, immune cell infiltration, or treatment effects. The study was carried out in a clinical research facility that allowed for standardized image and measurement acquisition (i.e., light conditions and operators) in a structured manner, allowing for side-by-side comparisons of techniques. The dermatoscopic follow-up functionality allowed for exact traceability of location throughout the study (Figure S3, http://links.lww.com/LGT/A299), and the biopsy location aligned with the obtained noninvasive measurements.

Performing a data-rich pilot trial in vulvar disease has inherently resulted in modest sample sizes. Unfortunately, not all intended vHSIL and no vulvar squamous cell carcinoma (VSCC) patients could be recruited, mainly due to the short and emotionally intense period between diagnosis and timely scheduled treatment. This pilot trial had intended to include VSCC patients to portray the complete pathway from healthy vulvar skin to VSCC. This statement could be expanded to all patients visiting the vulvar consultation office with a variety of vulvar diseases, including differentiated vulvar intraepithelial neoplasia (dVIN), because the possible discriminative nature of promising characteristics should be validated in a practical sample. In addition, to contribute a diverse and representative study population, the field should aim to include women with all Fitzpatrick skin types because dermatoses can be phenotypically distinct on darker skin types than those included in this patient sample. Finally, 7/10 histological assessments of LS were incongruent with the clinical diagnosis. This discrepancy is not considered a limitation because LS is primarily a clinical diagnosis, but highlights the heterogeneity of this vulvar disease. Lichen sclerosus can present heterogeneously in the clinic (with atrophy, fibrosis, or inflammation). This clinical, morphological, and histological variability could influence the findings of our study and should be considered in further interpretations.

Dermatoscopy is an integrative part of the dermatologists' evaluation of potentially malignant cutaneous lesions.^4^ However, the evaluation of vulvar disease using sophisticated imaging devices is uncommon in daily vulvar clinic or gynecological practice. An expanding catalogue of reports describe dermatoscopy for vulvar lesions, but a well-established and structured approach of image acquisition and reporting remains lacking.^5,6,24,25^ Observations in intraepithelial neoplasia have been summarized in a recent review, although the overview does not include stratification for sex or disease subtypes (i.e., vHSIL or HPV-independent differentiated vulvar intraepithelial neoplasia).^26^ The currently recognized characteristics include red to white structureless areas in addition to presence of dotted, glomerular, and linear vessels. Gray-brownish dots have been described in pigmented intraepithelial neoplasia lesions. Our dermatoscopy results in vHSIL concur with the literature, although the modest patient population restricts further comparisons. Features identified in vHSIL could also be found in LS or healthy controls, rendering none of the identified characteristics disease-specific. The only distinctive feature in our study was warty structures in vHSIL, but this adds little clinical value because this is clear on visual inspection. Plus, many vulvar diagnoses may present as warty structures, such as condylomata acuminata or papillomatosis.^12^

On D-OCT, we found an increased blood flow in nonlesional vHSIL compared with healthy vulvar skin. This observation may be due to a more extensively inflamed vulvar area besides the clinically observable vHSIL lesion(s). This implies that nonlesional, apparently healthy, vulvar skin of vHSIL patients should not be considered a valid healthy control, that is, within-patient controls can cause potential confounding. The same notion applies for nonlesional LS skin, which can appear without clinical signs of LS but in fact may comprise preclinical diseased vulvar skin. Our conclusions may have been influenced by the small cohort and potential artifacts from warty lesional structures on the blood flow measurements. Histologically, acanthosis is a well-known feature of vHSIL.^23^ Structural OCT analysis noninvasively found a thicker epidermis for lesional vHSIL than healthy vulvar skin, as reported once previously.^12^ However, the OCT software algorithm is inadequate for epidermal thickness measurements, most likely due to anatomically irregular vulvar structures. Unfortunately, manual measurements are too time-consuming. Improvement of the software algorithm for vulvar skin would be required to make this OCT parameter applicable for practical implementation.

Several reports have described dermatoscopic features of LS.^17,25,27,28^ A recent review summarized dermatoscopic features of LS, which reportedly appears with structureless areas, red globules in a white background with a decrease, or “desertification”, of vessels.^26^ Our observations are in line with these results, with the notable exception with regard to vascular changes in a number of cases. We found more pronounced vasculature primarily consisting of thick and thin arborizing vessels in approximately 40% of LS cases. Generally, these patients presented clinically with a loss of vulvar architecture. Literature is yet undecided whether dermatoscopic vascular patterns could correlate to disease duration.^18,27^

These newly described dermatoscopic thick and thin arborizing vessels concur with established histological features of hyalinized, stiff vessels in the dermis of LS.^20,21^ These stiff vessels translated into the observed increase in blood flow in lesional LS vulvar skin, as measured by D-OCT. We hypothesize that this could be the result of sclerosis and damage to the connective tissue in LS, affecting the microvasculature of the dermis.^29^ The observed increase in dermal blood flow in genital LS has not previously been objectified by D-OCT, but are in agreement with previous descriptions using laser Doppler and in 3 patients with extragenital LS.^30,31^ Histologically, the vulvar epidermis in LS is thinner compared with healthy vulvar skin.^21^ We confirm epidermal thinning in LS numerically and morphologically using noninvasive, structural OCT measurements.

## CONCLUSIONS

This study describes a structured, prospective approach to identify sophisticated imaging methods for vHSIL and LS. Using dermatoscopy and D-OCT, we described potential characteristics to aid differentiation of diseased from healthy vulvar skin. Dermatoscopy is a promising tool that may facilitate clinical recognition and follow-up of vHSIL and LS after expansion of patient groups and clinical validation. Vulvar biopsies can be obtained on a limited basis, whereas noninvasive techniques can be used repeatedly, minimizing patient burden. The step to clinical integration of D-OCT is considered inappropriate at this stage due to the suboptimal algorithms and remaining questions on the applicability in clinical practice. Imaging techniques should always be preceded by visual examination to establish a clinical differential diagnosis. Our findings require confirmation in larger, more diverse cohorts including suspicious lesions of the vulva over time before implementation in the vulvar clinic.

## Supplementary Material

SUPPLEMENTARY MATERIAL

## References

[1] Lockhart J Gray NM Cruickshank ME. The development and evaluation of a questionnaire to assess the impact of vulval intraepithelial neoplasia: a questionnaire study. *BJOG* 2013;120:1133–43. 23573981 10.1111/1471-0528.12229

[2] Jamieson A Tse SS Proctor L, . A scoping review of treatment outcome measures for vulvar intraepithelial neoplasia. *J Low Genit Tract Dis* 2022;26:328–38. 36074136 10.1097/LGT.0000000000000698

[3] Kruizinga MD Stuurman FE Exadaktylos V, . Development of novel, value-based, digital endpoints for clinical trials: a structured approach toward fit-for-purpose validation. *Pharmacol Rev* 2020;72:899–909. 32958524 10.1124/pr.120.000028

[4] Ring C Cox N Lee JB. Dermatoscopy. *Clin Dermatol* 2021;39:635–42. 34809768 10.1016/j.clindermatol.2021.03.009

[5] Ronger-Savle S Julien V Duru G, . Features of pigmented vulval lesions on dermoscopy. *Br J Dermatol* 2011;164:54–61. 20846309 10.1111/j.1365-2133.2010.10043.x

[6] Ferrari A Zalaudek I Argenziano G, . Dermoscopy of pigmented lesions of the vulva: a retrospective morphological study. *Dermatology* 2011;222:157–66. 21311169 10.1159/000323409

[7] Cengiz FP Emiroglu N Wellenhof RH. Dermoscopic and clinical features of pigmented skin lesions of the genital area. *An Bras Dermatol* 2015;90:178–83. 10.1590/abd1806-4841.20153294 PMC4371665 25830986

[8] Fujimoto J Swanson E. The development, commercialization, and impact of optical coherence tomography. *Invest Ophthalmol Vis Sci* 2016;57:OCT1–OCT13. 27409459 10.1167/iovs.16-19963 PMC4968928

[9] Marschall S Sander B Mogensen M, . Optical coherence tomography-current technology and applications in clinical and biomedical research. *Anal Bioanal Chem* 2011;400:2699–720. 21547430 10.1007/s00216-011-5008-1

[10] Wan B Ganier C Du-Harpur X, . Applications and future directions for optical coherence tomography in dermatology. *Br J Dermatol* 2021;184:1014–22. 32974943 10.1111/bjd.19553

[11] Escobar PF Belinson JL White A, . Diagnostic efficacy of optical coherence tomography in the management of preinvasive and invasive cancer of uterine cervix and vulva. *Int J Gynecol Cancer* 2004;14:470–4. 15228420 10.1111/j.1048-891x.2004.14307.x

[12] Wessels R de Bruin DM Faber DJ, . Optical coherence tomography in vulvar intraepithelial neoplasia. *J Biomed Opt* 2012;17:116022. 23214182 10.1117/1.jbo.17.11.116022

[13] Xu L Ma Q Lin S, . Study on the application and imaging characteristics of optical coherence tomography in vulva lesions. *Sci Rep* 2022;12:3659. 35256649 10.1038/s41598-022-07634-1 PMC8901679

[14] Kirillin M Motovilova T Shakhova N. Optical coherence tomography in gynecology: a narrative review. *J Biomed Opt* 2017;22:1–9. 10.1117/1.JBO.22.12.121709 29210220

[15] Rissmann R Moerland M van Doorn MBA. Blueprint for mechanistic, data-rich early phase clinical pharmacology studies in dermatology. *Br J Clin Pharmacol* 2020;86:1011–4. 32253783 10.1111/bcp.14293 PMC7256123

[16] Hehir M Du Vivier A Eilon L, . Investigation of the pharmacokinetics of clobetasol propionate and clobetasone butyrate after a single application of ointment. *Clin Exp Dermatol* 1983;8:143–51. 6851236 10.1111/j.1365-2230.1983.tb01758.x

[17] Borghi A Corazza M Minghetti S, . Dermoscopic features of vulvar lichen sclerosus in the setting of a prospective cohort of patients: new observations. *Dermatology* 2016;232:71–7. 26574744 10.1159/000439198

[18] Borghi A Virgili A Corazza M. Dermoscopy of inflammatory genital diseases: practical insights. *Dermatol Clin* 2018;36:451–61. 30201154 10.1016/j.det.2018.05.013

[19] Themstrup L Welzel J Ciardo S, . Validation of dynamic optical coherence tomography for non-invasive, in vivo microcirculation imaging of the skin. *Microvasc Res* 2016;107:97–105. 27235002 10.1016/j.mvr.2016.05.004

[20] Morrel B Ewing-Graham PC van der Avoort IAM, . Structured analysis of histopathological characteristics of vulvar lichen sclerosus in a juvenile population. *Hum Pathol* 2020;106:23–31. 32971127 10.1016/j.humpath.2020.09.003

[21] Regauer S Liegl B Reich O. Early vulvar lichen sclerosus: a histopathological challenge. *Histopathology* 2005;47:340–7. 16178888 10.1111/j.1365-2559.2005.02209.x

[22] Trietsch MD Peters AA Gaarenstroom KN, . Spindle cell morphology is related to poor prognosis in vulvar squamous cell carcinoma. *Br J Cancer* 2013;109:2259–65. 24064972 10.1038/bjc.2013.563 PMC3798963

[23] Hoang LN Park KJ Soslow RA, . Squamous precursor lesions of the vulva: current classification and diagnostic challenges. *Pathology* 2016;48:291–302. 27113549 10.1016/j.pathol.2016.02.015 PMC5518939

[24] Oakley A. Dermatoscopic features of vulval lesions in 97 women. *Australas J Dermatol* 2016;57:48–53. 25754966 10.1111/ajd.12298

[25] Corazza M Borghi A Minghetti S, . Dermoscopy of isolated syringoma of the vulva. *J Am Acad Dermatol* 2017;76:S37–9. 28087025 10.1016/j.jaad.2016.06.009

[26] Lacarrubba F Borghi A Verzì AE, . Dermoscopy of genital diseases: a review. *J Eur Acad Dermatol Venereol* 2020;34:2198–207. 32531092 10.1111/jdv.16723

[27] Larre Borges A Tiodorovic-Zivkovic D Lallas A, . Clinical, dermoscopic and histopathologic features of genital and extragenital lichen sclerosus. *J Eur Acad Dermatol Venereol* 2013;27:1433–9. 22646723 10.1111/j.1468-3083.2012.04595.x

[28] Shim W-H Jwa S-W Song M, . Diagnostic usefulness of dermatoscopy in differentiating lichen sclerous et atrophicus from morphea. *J Am Acad Dermatol* 2012;66:690–1. 22421117 10.1016/j.jaad.2011.06.042

[29] Sirotkina MA Potapov AL Vagapova NN, . Multimodal optical coherence tomography: imaging of blood and lymphatic vessels of the vulva. *Sovrem Tehnol v Med* 2019;11:26–31.

[30] Saravanamuthu J Seifalian AM Reid WM, . A new technique to map vulva microcirculation using laser Doppler perfusion imager. *Int J Gynecol Cancer* 2003;13:812–8. 14675318 10.1111/j.1525-1438.2003.13047.x

[31] Ring HC Mogensen M Hussain AA, . Imaging of collagen deposition disorders using optical coherence tomography. *J Eur Acad Dermatol Venereol* 2015;29:890–8. 25178655 10.1111/jdv.12708

