# Supplementary material for: Dermatoscopy and Optical Coherence Tomography in Vulvar High-Grade Squamous Intraepithelial Lesions and Lichen Sclerosus: A Prospective Observational Trial
Source: J Low Genit Tract Dis. 2023 Mar 16;27(3):255–61. doi: 10.1097/LGT.0000000000000731 (PMC10309090; doi:10.1097/LGT.0000000000000731)
Supplement: SUPPLEMENTARY MATERIAL [file jlgtd-27-255-s002.docx]

# Title

# Dermatoscopy and optical coherence tomography in vulvar HSIL and lichen sclerosus: a prospective observational trial

## Running title

Dermatoscopy and D-OCT for vHSIL and LS

## PRECIS

This feasibility study presents dermatoscopy and D-OCT as easy-to-use, well-tolerated and non-invasive imaging tools aiding recognition of vHSIL and lichen sclerosus.

## ABSTRACT

**Objective**: To examine potential discriminatory characteristics of dermatoscopy and dynamic optical coherence tomography (D-OCT) on vulvar high-grade squamous intraepithelial lesions (vHSIL) and lichen sclerosus (LS) compared to healthy vulvar skin.

**Methods:** A prospective observational clinical trial was performed in 10 healthy volunteers, 5 vHSIL and 10 LS patients. Non-invasive imaging measurements using dermatoscopy and D-OCT were obtained at several time-points, including lesional and non-lesional vulvar skin. Morphologic features of vHSIL and LS were compared to healthy controls. Epidermal thickness and blood flow were determined using D-OCT. Patients reported tolerability of each study procedure, including reference vulvar biopsies. The main outcome measures were Feasibility and tolerability of imaging modalities, dermatoscopy and OCT characteristics, OCT epidermal thickness and D-OCT dermal blood flow.

**Results**: The application of dermatoscopy and D-OCT is feasible and tolerable. In vHSIL, dermatoscopic warty structures were present. In LS, sclerotic areas and arborizing vessels were observed. Structural OCT in the vulvar area aligned with histology for hyperkeratosis and dermal-epidermal junction visualisation. Currently, the OCT algorithm is unable to calculate the epidermal thickness of the uneven vulvar area. D-OCT showed statistically significant increased blood flow in LS patients (mean ±SD 0.053 ±0.029) to healthy controls (0.040 ±0.012, p=0.0024).

**Conclusions**: The application of dermatoscopy and D-OCT is feasible and tolerable in vHSIL and LS patients. Using dermatoscopy and D-OCT, we describe potential characteristics to aid differentiation of diseased from healthy vulvar skin, which could complement clinical assessments.

**Keywords**: dermatoscopy, D-OCT, lichen sclerosus, vulvar HSIL

## Total word count: 3050

## INTRODUCTION

Inadequate clinical recognition and delayed or inadequate treatment of vulvar high-grade intraepithelial lesions (vHSIL) and lichen sclerosus (LS) can have considerable physical, sexual and psychological impact.^1^ The diagnostic process and disease follow-up of these vulvar diseases should be improved by objective and non-invasive, disease-specific biomarkers.^2^ A prerequisite in biomarker validation is its ability to discriminate healthy from diseased tissue.^3^

Examples of potential novel techniques are dermatoscopy and dynamic optical coherence tomography (D-OCT). Dermatoscopy is routinely applied by dermatologists as adjunctive tool to ameliorate subsurface structure visualization and pattern identification to aid diagnosis of melanoma, basal cell carcinoma and other cutaneous disorders.^4^ Its application on the vulvar area is currently limited to research purposes of vulvar pigmented lesions.^5–7^ D-OCT is a non-invasive imaging technique that provides real-time cross-sectional images of biological structures. It has been incorporated in the daily ophthalmology practice for diagnosis of retinal diseases.^8^ D-OCT has been applied as a research tool in dermatology for characterisation of non-melanoma skin cancer.^9,10^ In gynaecology, a few studies in cervical, vulvar and ovarian tissue suggest potential for D-OCT to differentiate between healthy and (pre)malignant tissue of epithelial origin.^11–14^

The study objective was to examine and describe potential discriminatory characteristics of dermatoscopy and D-OCT on premalignant vulvar skin compared to healthy vulvar skin. Therefore, we examined dermatoscopy and D-OCT on vHSIL and LS patients and healthy controls.

## METHODS

A prospective, healthy volunteer-matched, single-centre trial conducted at the Centre for Human Drug Research, Leiden, The Netherlands, was performed from February 2021 to October 2021. The Declaration of Helsinki was the guiding principle for trial execution. The study was approved by an independent medical-ethics committee “Medisch-Ethische Toetsingscommissie Leiden Den Haag Delft” and registered in “Nederlands Trial Register” (NL73964.058.20) and “EudraCT” (2020-002201-2). All subjects provided written informed consent before participation. These imaging results are part of a multi-modal pilot study investigating research methods to identify biomarkers that could improve vulvar disease identification and therapeutic response monitoring (Figure S1, <http://links.lww.com/LGT/A297>).^15^

### Study design and subjects

In total, 25 women, aged 25-95 with a body mass index (BMI) <30 kg/m^2^ were included: 5 vHSIL patients (≥1 sharply margined histologically confirmed vHSIL lesion ≥15mm), 10 LS patients (clinical and/or histological diagnosis confirmation) and 10 healthy controls (confirmed absence of vulvar disease). Main exclusion criteria were significant other diseases, pregnancy, other vulvar conditions, immunocompromised state, sexually transmitted disease, AIDS or hepatitis. For standardization, wash-out for topically applied products on the vulvar area was ≥14 days.^16^

All subjects visited the clinical research department on Day 0 (0h = baseline, 3h and 6h), Day 1 and Day 7 (Figure S2, <http://links.lww.com/LGT/A298>). LS patients also visited the clinic on Day 21 and 35, as follow-up for a 4-week standard of care treatment with corticosteroid ointment clobetasol 0.05% (Dermovate, GlaxoSmithKline, Brentford, UK) starting at Day 8. At each visit, clinical assessments and non-invasive imaging measurements were performed. Biopsies were obtained at Day 0 for all subjects and on Day 35 for LS patients.

### Anogenital examination

Examination of the anogenital region and study procedures were performed in a gynaecological chair. All patients were assessed by trained physicians (BH and LP) and discussed with an oncological gynaecologist specialized in vulvar disease (MvP) at baseline. Although LS has a heterogeneous clinical presentation, we opted out of further stratification due to low patient numbers and the exploratory scope of our study. All procedures were performed on selected target areas, including a lesional and non-lesional site for all patients.

### Imaging

#### Dermatoscopy on the vulvar area

Macroscopic dermatoscopic images of the vulvar surface were obtained using a FotoFinder Medicam 1000 with the Bodystudio ATBM (FotoFinder Systems GmbH, Bad Birnbach, Germany) for photo analysis and documentation (Figure S3, <http://links.lww.com/LGT/A299>). Microscopic images were obtained with a D-Scope IV dermatoscopy lens with polarized light and analysed using FotoFinder universe. Dermatoscopy includes a follow-up photo documentation function.

##### Microscopic characteristics

Individual dermatoscopic characteristic or a set of characteristics were exploratorily assessed for discriminatory potential for vulvar diseases. An expert dermatologist fully blinded for patient type (CH) scored characteristics in decoded microscopic images. The characteristics included: colour of the skin (red, pink, yellow, grey, brown, white or other); vessel density or concentration (increased, normal, decreased or invisible) and vessel concentration (dotted, hairpin, linear, linear serpentine, thin/thick arborizing, thick root-like, other or not visible). The presence or absence of scales, ecchymoses, purpura, yellow-white structureless areas, white circles, peppering, comedo-like openings, ulceration and warty structures were reported, as previously described in literature of dermatoscopy characteristics of genital lesions.^17,18^ In total, 85 photos were scored (15 vHSIL, 40 LS and 30 healthy volunteers) obtained at Day 0, 7 and 35.

#### D-OCT

Skin morphology analysis up to a depth of 1 mm was performed by D-OCT using the Vivosight Dx (Michelson Diagnostics Ltd., United Kingdom) (Figure S4, <http://links.lww.com/LGT/A300>). Scans with artefacts due to movement were excluded from analysis and directly retaken. Data were stored and analysed using VivoSight and VivoTools version 4.15. Qualitative assessments were performed by three trained OCT operators (BH, LP and WV).

##### Epidermal thickness

Epidermal thickness was determined using algorithms incorporated in the software. Manual epidermal thickness analyses were performed with ImageJ (version Java 1.8.0_172, Bethesda, Maryland, USA). Using a self-generated macro, three consecutive vertical lines were drawn for the epidermal layer per scan. The mean, SD, minimum and maximum epidermal thickness were determined per set of 120 consecutive scans per patient. Baseline and post-treatment (LS) scans were analysed manually for epidermal thickness due to the exploratory and time-consuming nature of manual calculations.

##### Blood flow

D-OCT blood flow was determined using the algorithms incorporated in the software. The quantification of the blood flow was based on the average speckle signal returning at the detector at dermal depth from 0.10 to 0.35mm to reduce contortions from artefacts.^19^

### Histological analysis

Vulvar tissue samples were obtained using a 4mm punch biopsy acquired by trained physicians (BH, LP and MvP) at the end of Day 0. The skin was anesthetised using subcutaneous lidocaine prior to the procedure. The obtained biopsies were stained for hematoxylin and eosin (HE) by the Erasmus Medical Centre (EMC, Rotterdam, The Netherlands) following clinical protocols. Slides were scored by a dermatopathologist (JD). Dysplasia was assessed by the epidermal levels of atypia and scored as warty and/or basaloid types. LS was diagnosed by histological characteristics.^20,21^ Inno-LiPa HPV Genotyping Extra (Eurofins NMDL-LCPL, Rijswijk, The Netherlands) was used for HPV typing. ^22^

### Patient reported outcomes

The ‘burdensome questionnaire’ comprised of 100 mm lines which the patient completed for each study procedure, ranging 0 mm “no burden at all” to 100 mm “the most burdensome procedure possible”. The e-diary (Promasys® ePRO platform) with a reminder and photograph function (with corresponding timestamps) monitored at-home drug compliance.

### Statistical analysis

Dermatoscopic observations were summarized and shown descriptively. Differences of D-OCT between patient groups were tested using Mann-Whitney U Test on baseline averages of two groups (vHSIL, LS or healthy). A paired, two-tailed t-test was performed comparing mean D-OCT values pre- to post-treatment. Differences for the burdensome questionnaire were analysed using a paired student’s t-test comparing dermatoscopy and D-OCT to the biopsy procedure. The analyses were computed in SAS 9.4 and GraphPad version 9.3.1.

## RESULTS

In total, 25 women (5 patients with vHSIL and 10 patients with LS and 10 healthy controls) were enrolled and finished the study (Table S1, <http://links.lww.com/LGT/A304>). Fitzpatrick skin-type ranged from I-III. Menopausal status was equally distributed among groups.

**Dermatoscopy**

Vulvar skin of a representative subject of each cohort (vHSIL, LS and healthy controls) captured by dermatoscopy is presented in a macroscopic overview and a microscopic image (Figure S5, <http://links.lww.com/LGT/A301>).

##### Microscopic characteristics

Examples of dermatoscopic characteristics are shown in Figure 1 and the frequency of observations per group are summarized in Figure 2. The most prominent characteristic of vHSIL were warty structures (4/5), which could be accompanied by some scales and peppering. The colour of vHSIL skin was highly variable. Women with Fitzpatrick skin-type >III were could not be recruited, so colour findings may vary based on the analysed population. Vessels were present with dotted or linear vessel. LS typically showed white structureless areas (8/10) and/or increased vessel concentration (8/10), with arborizing and/or thick root-like vessel morphology. The vulvar skin of healthy controls was mostly yellow (8/10), with normal vessel pattern of dotted or linear vessels, sometimes accompanied by white circles (4/10). Occasionally white structureless areas or peppering was observed (3/10). No changes were observed in LS skin after 4-week clobetasol treatment (data not shown).

### D-OCT

##### Morphological characteristics

vHSIL is histologically characterized by hyperkeratosis and parakeratosis, acanthosis with club-shaped rete ridges, cytonuclear atypia, disorientation of individual epithelial cells and an intact basement membrane.^23^ Hyperkeratosis could be identified in the structural OCT image of a vHSIL lesion as hyperreflective stratum corneum. OCT shows the broadening of the epidermis in the club-shaped pattern associated with acanthotic vHSIL with an intact dermal-epidermal junction, as observed in histology (Figure 3A). LS is histologically characterized by hyperkeratosis, epidermal thinning with loss of the rete ridge pattern and dermal changes, including sclerosis.^20,21^ These changes can also be identified with OCT, especially the disorganized extracellular matrix reflecting dermal changes (Figure 3B). Nuclear and cellular changes cannot be visualized using OCT. We observed that resolution was occasionally lost under a hyperkeratotic and hyperreflective stratum corneum or sclerotic area. These OCT findings are pronounced in vHSIL and LS compared to healthy vulvar skin. Histologically, healthy vulvar skin has a normal epidermal thickness in the absence of characteristics observed in diseased vulvar skin. These features could also be visualized in OCT recordings (Figure 3C).

##### Epidermal thickness

In total 77.5% (vHSIL), 56.9% (LS) and 91.2% (healthy control) of the measurements using the incorporated algorithm failed, as an impossible epidermal thickness of 0 µm was reported . Therefore, manual epidermal thickness measurements were performed (Figure 4A+B). No significant differences in epidermal thickness were identified comparing lesional or non-lesional vHSIL to healthy controls. The epidermis (mean±SD) of pre-clobetasol lesional LS (0.13±0.10µm) was significantly thinner compared to healthy controls (0.19±0.06µm), p=0.0312. No differences were observed between pre- and post-clobetasol-treated LS (0.127±0.10µm vs 0.118±0.034, p=0.643).

##### Blood flow

At baseline, higher blood flow (mean±SD) was observed in non-lesional vHSIL (0.063 ±0.040) compared to lesional vHSIL (0.044±0.025), p=0.0255 (Figure 4C). No differences were detected between lesional vHSIL and healthy controls (0.040±0.017, p=0.347). Blood flow in non-lesional vHSIL skin differed significantly from healthy controls (0.063±0.040, p=0.0001). Blood flow was significantly higher in pre-treatment lesional LS (0.053±0.029) compared to non-lesional LS (0.034±0.019, p<0.0001) and healthy controls (0.040±0.012, p=0.0024) (Figure 4D). Non-lesional LS did not differ significantly compared to healthy controls (p=0.077). No differences were observed between pre- and post-clobetasol-treated LS (0.057±0.042) (p=0.532). Blood flow measurements fluctuated over time (Day 0, 2, 8, 22 and 36), depending on sample location (lesional vs non-lesional) (Figure S6, <http://links.lww.com/LGT/A302>).

### Histological analysis

All clinical diagnoses of non-lesional and lesional skin of vHSIL, non-lesional LS and healthy controls were confirmed in biopsy (Table S2, <http://links.lww.com/LGT/A305>). The biopsies of lesional LS were classified as LS in 3/10 cases. The remaining were classified as normal vulvar skin with inflammatory reactive changes, although clinical LS diagnosis had been confirmed by a specialized gynaecologist (MvP) prior to enrolment. Positivity for HPV16 was identified in 4/5 lesional vHSIL biopsies. One lesional LS biopsy tested positive for HPV type 53, while no HPV was found in non-lesional or healthy control biopsies.

### Patient-friendliness and treatment compliance

All imaging methods applied in this study were considered mildly burdensome and therefore patient friendly. Vulvar biopsy was considered substantially more burdensome than all non-invasive imaging procedures, with mean scores >20 mm (Figure S7, <http://links.lww.com/LGT/A303>). Treatment compliance was 99%.

## DISCUSSION

This exploratory study shows that application of dermatoscopy and D-OCT is feasible and tolerable in vHSIL and LS patients. The most prominent finding with dermatoscopy was presence of sclerotic areas and arborizing vessels in LS and warty structures for vHSIL. Structural OCT images could be aligned for both diseases with histology. A novel finding in this study was the increased blood flow measured by the algorithm of the D-OCT in vulvar LS compared to healthy tissue. Epidermal thickness determination by OCT should be considered for research purposes only at this stage.

The main strength is the prospective trial design in vulvar patients and healthy controls including within-subject lesional and non-lesional control. Including only within-patient “healthy” control sites can be invalid, as the vulvar tissue may be affected by scarring, immune cell infiltration or treatment effects. The study was carried out in a clinical research facility that allowed for standardized image and measurement acquisition (i.e. light conditions and operators) in a structured manner, allowing for side-by-side comparisons of techniques. The dermatoscopic follow-up functionality allowed for exact traceability of location throughout the study (Figure S3, <http://links.lww.com/LGT/A299>) and the biopsy location aligned with the obtained non-invasive measurements.

Performing a data-rich pilot trial in vulvar disease has inherently resulted in a modest sample sizes. Unfortunately, not all intended vHSIL and no vulvar squamous cell carcinoma (VSCC) patients could be recruited, mainly due to the short and emotionally intense period between diagnosis and timely scheduled treatment. This pilot trial had intended to include VSCC patients to portray the complete pathway from healthy vulvar skin to VSCC. This statement could be expanded to all patients visiting the vulvar consultation office with a variety of vulvar diseases, including differentiated vulvar intraepithelial neoplasia (dVIN), as the possible discriminative nature of promising characteristics should be validated in a practical sample. In addition, to contribute a diverse and representative study population, the field should aim to include women with all Fitzpatrick skin-types, as dermatoses can be phenotypically distinct on darker skin types than those included in this patient sample. Finally, 7/10 histological assessments of LS were incongruent with the clinical diagnosis. This discrepancy is not considered a limitation, as LS is primarily a clinical diagnosis, but highlights the heterogeneity of this vulvar disease. LS can present heterogeneously in the clinic (with atrophy, fibrosis or inflammation). This clinical, morphological, and histological variability could influence the findings of our study and should be considered in further interpretations.

Dermatoscopy is an integrative part of the dermatologists’ evaluation of potentially malignant cutaneous lesions.^4^ However, the evaluation of vulvar disease using sophisticated imaging devices is uncommon in daily vulvar clinic or gynaecological practice. An expanding catalogue of reports describe dermatoscopy for vulvar lesions, but a well-established and structured approach of image acquisition and reporting remains lacking.^5,6,24,25^ Observations in intraepithelial neoplasia have been summarized in a recent review, although the overview does not include stratification for sex or disease subtypes (i.e. vHSIL or HPV-independent dVIN).^26^ The currently recognized characteristics include red to white structureless areas in addition to presence of dotted, glomerular and linear vessels. Grey-brownish dots have been described in pigmented intraepithelial neoplasia lesions. Our dermatoscopy results in vHSIL concur with literature, although the modest patient population restricts further comparisons. Features identified in vHSIL could also be found in LS or healthy controls, rendering none of the identified characteristics disease specific. The only distinctive feature in our study were warty structures in vHSIL, but this adds little clinical value as this is clear upon visual inspection. Plus, many vulvar diagnoses may present as warty structures, such as condylomata acuminata or papillomatosis.^12^

On D-OCT, we found an increased blood flow in non-lesional vHSIL compared to healthy vulvar skin. This observation may be due to a more extensively inflamed vulvar area, besides the clinically observable vHSIL lesion(s). This implies that non-lesional, apparently healthy, vulvar skin of vHSIL patients should not be considered a valid healthy control, i.e. within-patient controls can cause potential confounding. The same notion applies for non-lesional LS skin, which can appear without clinical signs of LS but in fact may comprise of pre-clinical diseased vulvar skin. Our conclusions may have been influenced by the small cohort and potential artefacts from warty lesional structures on the blood flow measurements. Histologically, acanthosis is a well-known feature vHSIL.^23^ Structural OCT analysis non-invasively found a thicker epidermis for lesional vHSIL than healthy vulvar skin, as reported once previously.^12^ However, the OCT software algorithm is inadequate for epidermal thickness measurements, most likely due to anatomically irregular vulvar structures. Unfortunately, manual measurements are too time-consuming. Improvement of the software algorithm for vulvar skin would be required to make this OCT parameter applicable for practical implementation.

Several reports have described dermatoscopic features of LS.^17,25,27,28^ A recent review summarized dermatoscopic features of LS, which reportedly appears with structureless areas, red globules in a white background with a decrease, or *desertification*, of vessels.^26^ Our observations are in line with these results, with the notable exception with regard to vascular changes in a number of cases. We found more pronounced vasculature primarily consisting of thick and thin arborizing vessels in approximately 40% of LS cases. Generally, these patients presented clinically with a loss of vulvar architecture. Literature is yet undecided whether dermatoscopic vascular patterns could correlate to disease duration.^18,27^

These newly described dermatoscopic thick and thin arborizing vessels concur with established histological features of hyalinized, stiff vessels in the dermis of LS.^20,21^ These stiff vessels translated into the observed increase in blood flow in lesional LS vulvar skin, as measured by D-OCT. We hypothesize that this could be the result of sclerosis and damage to the connective tissue in LS, affecting the microvasculature of the dermis.^29^ The observed increase in dermal blood flow in genital LS has not previously been objectified by D-OCT, but are in agreement with previous descriptions using Laser Doppler and in three patients with extragenital LS.^30,31^ Histologically, the vulvar epidermis in LS is thinner compared to healthy vulvar skin.^21^ We confirm epidermal thinning in LS numerically and morphologically using non-invasive structural OCT measurements.

## CONCLUSION

This study describes a structured, prospective approach to identify sophisticated imaging methods for vHSIL and LS. Using dermatoscopy and D-OCT, we described potential characteristics to aid differentiation of diseased from healthy vulvar skin. Dermatoscopy is a promising tool that may facilitate clinical recognition and follow-up of vHSIL and LS after expansion of patient groups and clinical validation. Vulvar biopsies can be obtained on a limited basis, whilst non-invasive techniques can be used repeatedly, minimizing patient burden. The step to clinical integration of D-OCT is considered inappropriate at this stage due to the suboptimal algorithms and remaining questions on the applicability in clinical practice. Imaging techniques should always be preceded by visual examination to establish a clinical differential diagnosis. Our findings require confirmation in larger, more diverse cohorts including suspicious lesions of the vulva over time before implementation in the vulvar clinic.

ABBREVIATIONS AND ACRONYMS

D-OCT: Dynamic optical coherence tomography

dVIN: Differentiated vulvar intraepithelial neoplasia

LS: Lichen sclerosus

vHSIL: Vulvar high-grade squamous intraepithelial lesions

## VSCC: Vulvar squamous cell carcinoma

## REFERENCES (Max 30)

1. Lockhart J, Gray N, Cruickshank M. The development and evaluation of a questionnaire to assess the impact of vulval intraepithelial neoplasia: a questionnaire study. *BJOG-An Int J Obstet Gynaecol*. 2013;120(9):1133-1143. doi:10.1111/1471-0528.12229

2. Jamieson A, Tse SS, Proctor L, Sadownik LA. A Scoping Review of Treatment Outcome Measures for Vulvar Intraepithelial Neoplasia. *J Low Genit Tract Dis*. 2022;26(4):328-338. doi:10.1097/LGT.0000000000000698

3. Kruizinga MD, Stuurman FE, Exadaktylos V, et al. Development of Novel, Value-Based, Digital Endpoints for Clinical Trials: A Structured Approach Toward Fit-for-Purpose Validation. *Pharmacol Rev*. 2020;72(4):899-909. doi:10.1124/PR.120.000028

4. Ring C, Cox N, Lee JB. Dermatoscopy. *Clin Dermatol*. 2021;39(4):635-642. doi:10.1016/J.CLINDERMATOL.2021.03.009

5. Ronger-Savle S, Julien V, Duru G, Raudrant D, Dalle S, Thomas L. Features of pigmented vulval lesions on dermoscopy. *Br J Dermatol*. 2011;164(1):54-61. doi:10.1111/J.1365-2133.2010.10043.X

6. Ferrari A, Zalaudek I, Argenziano G, et al. Dermoscopy of pigmented lesions of the vulva: a retrospective morphological study. *Dermatology*. 2011;222(2):157-166. doi:10.1159/000323409

7. Cengiz FP, Emiroglu N, Hofmann Wellenhof R. Dermoscopic and clinical features of pigmented skin lesions of the genital area. *An Bras Dermatol*. 2015;90(2):178-183. doi:10.1590/ABD1806-4841.20153294

8. Fujimoto J, Swanson E. The Development, Commercialization, and Impact of Optical Coherence Tomography. *Invest Ophthalmol Vis Sci*. 2016;57(9):OCT1-OCT13. doi:10.1167/iovs.16-19963

9. Marschall S, Sander B, Mogensen M, Jørgensen TM, Andersen PE. Optical coherence tomography-current technology and applications in clinical and biomedical research. *Anal Bioanal Chem*. 2011;400(9):2699-2720. doi:10.1007/S00216-011-5008-1

10. Wan B, Ganier C, Du-Harpur X, et al. Applications and future directions for optical coherence tomography in dermatology. *Br J Dermatol*. 2021;184(6):1014-1022. doi:10.1111/BJD.19553

11. Escobar PF, Belinson JL, White A, et al. Diagnostic efficacy of optical coherence tomography in the management of preinvasive and invasive cancer of uterine cervix and vulva. *Int J Gynecol Cancer*. 2004;14(3):470-474. doi:10.1136/IJGC-00009577-200405000-00008

12. Wessels R, Bruin DM de, Faber DJ, et al. Optical coherence tomography in vulvar intraepithelial neoplasia. *https://doi.org/101117/1JBO1711116022*. 2012;17(11):116022. doi:10.1117/1.JBO.17.11.116022

13. Xu L, Ma Q, Lin S, et al. Study on the application and imaging characteristics of optical coherence tomography in vulva lesions. *Sci Rep*. 2022;12(1):3659. doi:10.1038/S41598-022-07634-1

14. Kirillin M, Motovilova T, Shakhova N. Optical coherence tomography in gynecology: a narrative review. *J Biomed Opt*. 2017;22(12):1. doi:10.1117/1.JBO.22.12.121709

15. Rissmann R, Moerland M, van Doorn MBA. Blueprint for mechanistic, data-rich early phase clinical pharmacology studies in dermatology. *Br J Clin Pharmacol*. Published online 2020. doi:10.1111/bcp.14293

16. Hehir M, Vivier A DU, Eilon L, Danie MJ, Shenoy EVB. Investigation of the pharmacokinetics of clobetasol propionate and clobetasone butyrate after a single application of ointment. *Clin Exp Dermatol*. 1983;8(2):143-151. doi:10.1111/j.1365-2230.1983.tb01758.x

17. Borghi A, Corazza M, Minghetti S, Bianchini E, Virgili A. Dermoscopic Features of Vulvar Lichen Sclerosus in the Setting of a Prospective Cohort of Patients: New Observations. *Dermatology*. 2016;232(1):71-77. doi:10.1159/000439198

18. Borghi A, Virgili A, Corazza M. Dermoscopy of Inflammatory Genital Diseases: Practical Insights. *Dermatol Clin*. 2018;36(4):451-461. doi:10.1016/J.DET.2018.05.013

19. Themstrup L, Welzel J, Ciardo S, et al. Validation of Dynamic optical coherence tomography for non-invasive, in vivo microcirculation imaging of the skin. *Microvasc Res*. 2016;107:97-105. doi:10.1016/j.mvr.2016.05.004

20. Morrel B, Ewing-Graham PC, van der Avoort IAM, Pasmans SGMA, Damman J. Structured analysis of histopathological characteristics of vulvar lichen sclerosus in a juvenile population. *Hum Pathol*. 2020;106:23-31. doi:10.1016/J.HUMPATH.2020.09.003

21. Regauer S, Liegl B, Reich O. Early vulvar lichen sclerosus: a histopathological challenge. *Histopathology*. 2005;47(4):340-347. doi:10.1111/J.1365-2559.2005.02209.X

22. Xu L, Padalko E, Oštrbenk A, Poljak M, Arbyn M. Clinical Evaluation of INNO-LiPA HPV Genotyping EXTRA II Assay Using the VALGENT Framework. *Int J Mol Sci*. 2018;19(9). doi:10.3390/IJMS19092704

23. Hoang LN, Park KJ, Soslow RA, Murali R. Squamous precursor lesions of the vulva: current classification and diagnostic challenges. *Pathology*. 2016;48(4):291-302. doi:10.1016/j.pathol.2016.02.015

24. Oakley A. Dermatoscopic features of vulval lesions in 97 women. *Australas J Dermatol*. 2016;57(1):48-53. doi:10.1111/ajd.12298

25. Corazza M, Borghi A, Minghetti S, Ferron P, Virgili A. Dermoscopy of isolated syringoma of the vulva. *J Am Acad Dermatol*. 2017;76(2):S37-S39. doi:10.1016/J.JAAD.2016.06.009

26. Lacarrubba F, Borghi A, Verzì AE, Corazza M, Stinco G, Micali G. Dermoscopy of genital diseases: a review. *J Eur Acad Dermatol Venereol*. 2020;34(10):2198-2207. doi:10.1111/jdv.16723

27. Larre Borges A, Tiodorovic-Zivkovic D, Lallas A, et al. Clinical, dermoscopic and histopathologic features of genital and extragenital lichen sclerosus. *J Eur Acad Dermatol Venereol*. 2013;27(11):1433-1439. doi:10.1111/j.1468-3083.2012.04595.x

28. Shim W-H, Jwa S-W, Song M, et al. Diagnostic usefulness of dermatoscopy in differentiating lichen sclerous et atrophicus from morphea. *J Am Acad Dermatol*. 2012;66(4):690-691. doi:10.1016/j.jaad.2011.06.042

29. Sirotkina MA, Potapov AL, Vagapova NN, et al. Multimodal optical coherence tomography: Imaging of blood and lymphatic vessels of the vulva. *Sovrem Tehnol v Med*. 2019;11(4):26-31. doi:10.17691/STM2019.11.4.03

30. Saravanamuthu J, Seifalian AM, Reid WM, Maclean AB. A new technique to map vulva microcirculation using laser Doppler perfusion imager. *Int J Gynecol Cancer*. 2003;13(6):812-818. doi:10.1111/J.1525-1438.2003.13047.X

31. Ring HC, Mogensen M, Hussain AA, et al. Imaging of collagen deposition disorders using optical coherence tomography. *J Eur Acad Dermatol Venereol*. 2015;29(5):890-898. doi:10.1111/jdv.12708

## TABLE AND FIGURE LEGENDS

**Figure 1.** Representative images showing examples of scored characteristics. A) Scales (LS) B) Small white circles (LS) C) Warty structure (vHSIL) D) Purpura (LS) E) White structureless areas (LS) F) Peppering (healthy vulva) G) Dotted vessels (vHSIL) H) Linear and hairpin vessels (LS) I) Thick and thin arborizing vessels (LS). Abbreviations: vHSIL = vulvar high-grade squamous intraepithelial lesion, LS = lichen sclerosus.

**Figure 2**. Percentage of observed characteristics per patient group (85 photos were scored: 15 vHSIL, 40 LS and 30 healthy volunteers) from the scoring of microscopic dermatoscopy images by a blinded dermatologist (CH). Abbreviations: vHSIL = vulvar high-grade squamous intraepithelial lesion, LS = lichen sclerosus, HV = healthy vulva.

**Figure 3:** Structural OCT recordings compared to aligned histological assessments of A+B) vHSIL, C+D) lichen sclerosus and E+F) healthy volunteers. Asterisks (*) indicate blood vessels. Abbreviations: OCT = dynamic optical coherence tomography, H&E = hematoxylin and eosin, vHSIL = vulvar high-grade squamous intraepithelial lesion; DEJ = dermal-epidermal junction; ECM = extracellular matrix, vHSIL = vulvar high-grade squamous intraepithelial lesion.

**Figure 4:** D-OCT epidermal thickness measurements (manually determined using ImageJ) and blood flow measurements (determined by incorporated algorithm). Measurements were assessed at baseline (D1H0) and, for LS only, post-treatment (D35). The mean and standard deviations are displayed for each group. A) Epidermal thickness in µm (y-axis) is plotted against measurements clustered per patient group (x-axis). B) The average blood flow measured between a skin depth of 0.10-0.35 µm at non-biopsy sites of vHSIL and LS subjects compared to healthy controls. The mean and standard deviations are displayed for each group, as well as individual data points. Blood flow in AU (y-axis) plotted against measurements clustered per patient group (x-axis). ns = p>0.05, * = p ≤ 0.05, ** = p ≤ 0.01, *** = p ≤ 0.001, **** = p ≤ 0.0001. Abbreviations: vHSIL = vulvar high-grade squamous intraepithelial lesion, LS = lichen sclerosus, HV = healthy vulva. L = lesional and NL = non-lesional skin.

**Figure S1:** Multi-modal biomarker research and potential domains to guide research in the vulvar field. Abbreviations: HPV = human papilloma virus.

**Figure S2.** Clinical study design. Healthy subjects followed the same regimen as HSIL and LS patients up until day 7, except for a biopsy of lesional vulvar skin. LS patients started clobetasol treatment at day 7 and had a FU-visits at day 21 and a final visit at day 35, including another lesional biopsy. Abbreviations: QD = once daily, vHSIL = vulvar high grade squamous intraepithelial lesion, LS = lichen sclerosus.

**Figure S3.** The follow-up functionality of the dermatoscope (Fotofinder) for both macroscopic and microscopic images. A) Overview pane with a macroscopic image wherein locations are added manually with turquoise arrows to indicate different locations (1-5). Follow-up of the macroscopic pictures is shown in the yellow dashed box and of the microscopic location sites in the blue dashed box. B) Microscopic image taking overview with the current micro image indicated at the green dashed box, the reference microscopic image (ghost function also available) at the red dashed box and the current image and a close op of the image location of interest at the purple dashed box.

**Figure S4.** Example of the image acquisition of the dynamic optical coherence tomography (D-OCT). A: 3D rendering of the imaged surface. B: live view of the imaged vulvar skin throughout the procedure. C: visualisation of a horizontal cross-section of the imaged vulvar skin, taken at 0.20mm as indicated by the green line in D. Blood vessels are shown in red. D: cross-sectional view of the imaged vulvar skin over 6mm. Blood vessels are shown in red.

**Figure S5.** Dermatoscopic macroscopic and microscopic images of the vulva. A-C shows the macroscopic 2D image. The black boxes indicate the imaging and biopsy locations. D-F shows the microscopic dermatoscopic images of the areas indicated by a black box. Abbreviations: vHSIL = vulvar high grade squamous intraepithelial lesion.

**Figure S6**: Average blood flow at Day o (0h, 3h and 6h), Day 1, Day 7 and Day 21+35 (LS only) for vHSIL, LS and HV subjects at a skin depth between 0.10-0.35. Abbreviations: vHSIL = vulvar high grade squamous intraepithelial lesion, LS = lichen sclerosus, HV = healthy vulva, D = Day, H = Hour, AU = arbitrary unit.

**Figure S7:** Mean (SD) patient rating on VAS scale answering the question “In your experience, how burdensome did you find the assessment?”, shown per patient group. Abbreviations: HV = healthy volunteer, vHSIL = vulvar HSIL, LS = Lichen sclerosus, OCT = optical coherence tomography, biopsy = vulvar biopsy. ** p≤0.0001.

**Table S1.** Baseline characteristics. Abbreviations: HPV = human papilloma virus.

**Table S2:** Overview of histological reporting, p16 and p53 staining per subject. MvP = Expert gynaecologist specialized in vulvar disease. JD = expert dermatopathologists. Abbreviations: vHSIL = vulvar HSIL, VIN3= Vulvar intraepithelial neoplasia type 3 (former term for vHSIL), LS = lichen sclerosus.
